# Supplementary material for: Recovery of the lumbopelvic movement and muscle recruitment patterns using motor control exercise program in people with chronic nonspecific low back pain: A prospective study
Source: PLoS One. 2021 Nov 18;16(11):e0259440. doi: 10.1371/journal.pone.0259440 (PMC8601576; doi:10.1371/journal.pone.0259440)
Supplement: S1 File — (PDF) [file pone.0259440.s001.pdf]

The Keele STarT Back Screening Tool

Patient name: ______________________________ Date: ____________

Thinking about the **last 2 weeks** tick your response to the following questions:

		Disagree<br>0	Agree<br>1
1	My back pain has **spread down my leg(s)** at some time in the last 2 weeks	☐	☐
2	I have had pain in the **shoulder** or **neck** at some time in the last 2 weeks	☐	☐
3	I have only **walked short distances** because of my back pain	☐	☐
4	In the last 2 weeks, I have **dressed more slowly** than usual because of back pain	☐	☐
5	It's not really safe for a person with a condition like mine to be physically active	☐	☐
6	**Worrying thoughts** have been going through my mind a lot of the time	☐	☐
7	I feel that **my back pain is terrible** and **it's never going to get any better**	☐	☐
8	In general I have **not enjoyed** all the things I used to enjoy	☐	☐

9. Overall, how **bothersome** has your back pain been in the **last 2 weeks**?

Not at all	Slightly	Moderately	Very much	Extremely
☐	☐	☐	☐	☐
0	0	0	1	1

Total score (all 9): ________________ **Sub Score (Q5-9):_____________**

© Keele University 01/08/07
Funded by Arthritis Research UK

The STarT Back Tool Scoring System

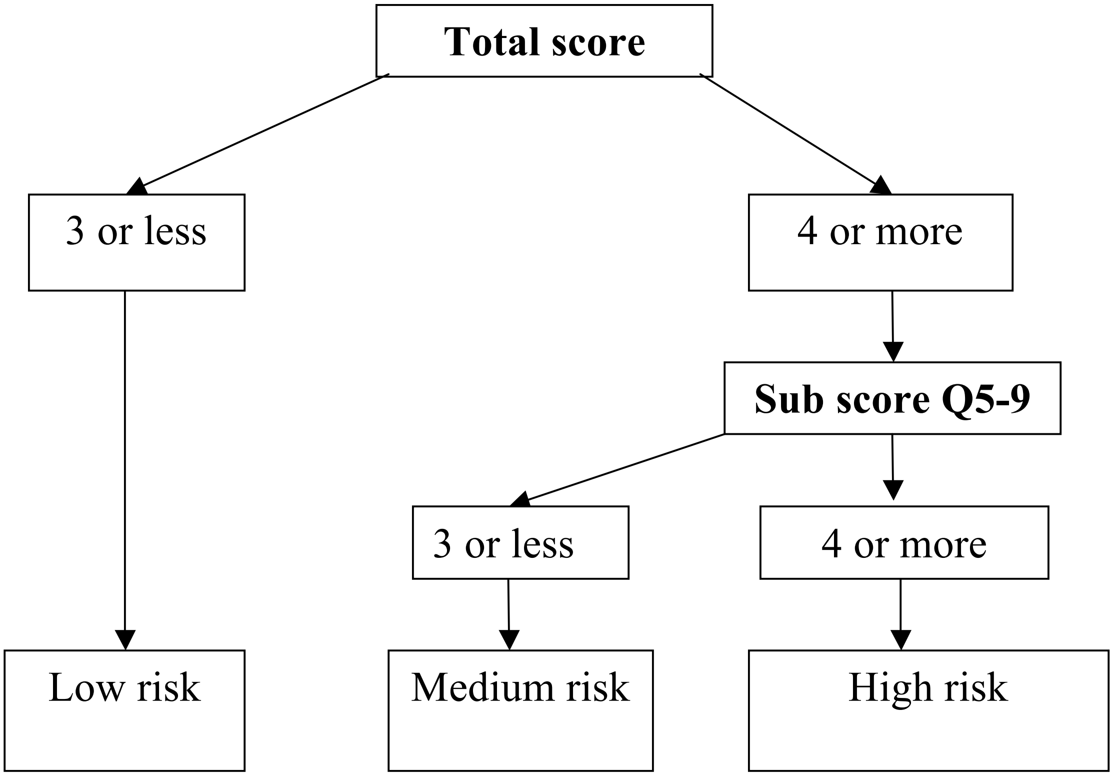

© Keele University 01/08/07
Funded by Arthritis Research UK

Tampa Scale for Kinesiophobia

(Miller , Kori and Todd 1991)

1 = strongly disagree
2 = disagree
3 = agree
4 = strongly agree

1. I'm afraid that I might injury myself if I exercise	1	2	3	4
2. If I were to try to overcome it, my pain would increase	1	2	3	4
3. My body is telling me I have something dangerously wrong	1	2	3	4
4. My pain would probably be relieved if I were to exercise	1	2	3	4
5. People aren't taking my medical condition seriously enough	1	2	3	4
6. My accident has put my body at risk for the rest of my life	1	2	3	4
7. Pain always means I have injured my body	1	2	3	4
8. Just because something aggravates my pain does not mean it is dangerous	1	2	3	4
9. I am afraid that I might injure myself accidentally	1	2	3	4
10. Simply being careful that I do not make any unnecessary movements is the safest thing I can do to prevent my pain from worsening	1	2	3	4
11. I wouldn't have this much pain if there weren't something potentially dangerous going on in my body	1	2	3	4
12. Although my condition is painful, I would be better off if I were physically active	1	2	3	4
13. Pain lets me know when to stop exercising so that I don't injure myself	1	2	3	4
14. It's really not safe for a person with a condition like mine to be physically active	1	2	3	4
15. I can't do all the things normal people do because it's too easy for me to get injured	1	2	3	4
16. Even though something is causing me a lot of pain, I don't think it's actually dangerous	1	2	3	4
17. No one should have to exercise when he/she is in pain	1	2	3	4

Reprinted from:
Pain, Fear of movement/(re) injury in chronic low back pain and its relation to behavioral performance, 62, Vlaeyen, J., Kole-Snijders A., Boeren R., van Eek H., 371.
Copyright (1995) with permission from International Association for the Study of Pain.

Scoring Information
Tampa Scale for Kinesiophobia
(Miller et al 1991)

A total score is calculated after inversion of the individual scores of items 4, 8, 12 and 16.

Reprinted from:
Pain, Fear of movement/(re) injury in chronic low back pain and its relation to behavioral performance, 62, Vlaeyen, J., Kole-Snijders A., Boeren R., van Eek H., 371.
Copyright (1995) with permission from International Association for the Study of Pain.

The Roland-Morris Disability Questionnaire

When your back hurts, you may find it difficult to do some of the things you normally do.

This list contains sentences that people have used to describe themselves when they have back pain. When you read them, you may find that some stand out because they describe you *today*.

As you read the list, think of yourself *today*. When you read a sentence that describes you today, put a tick against it. If the sentence does not describe you, then leave the space blank and go on to the next one. Remember, only tick the sentence if you are sure it describes you today.

1. I stay at home most of the time because of my back.

2. I change position frequently to try and get my back comfortable.

3. I walk more slowly than usual because of my back.

4. Because of my back I am not doing any of the jobs that I usually do around the house.

5. Because of my back, I use a handrail to get upstairs.

6. Because of my back, I lie down to rest more often.

7. Because of my back, I have to hold on to something to get out of an easy chair.

8. Because of my back, I try to get other people to do things for me.

9. I get dressed more slowly then usual because of my back.

10. I only stand for short periods of time because of my back.

11. Because of my back, I try not to bend or kneel down.

12. I find it difficult to get out of a chair because of my back.

13. My back is painful almost all the time.

14. I find it difficult to turn over in bed because of my back.

15. My appetite is not very good because of my back pain.

16. I have trouble putting on my socks (or stockings) because of the pain in my back.

17. I only walk short distances because of my back.

18. I sleep less well because of my back.

19. Because of my back pain, I get dressed with help from someone else.

20. I sit down for most of the day because of my back.

21. I avoid heavy jobs around the house because of my back.

22. Because of my back pain, I am more irritable and bad tempered with people than usual.

23. Because of my back, I go upstairs more slowly than usual.

24. I stay in bed most of the time because of my back.

<u>Note to users:</u>

This questionnaire is taken from: Roland MO, Morris RW. A study of the natural history of back pain. Part 1: Development of a reliable and sensitive measure of disability in low back pain. Spine 1983; 8: 141-144

The score of the RDQ is the total number of items checked – i.e. from a minimum of 0 to a maximum of 24.

It is acceptable to add boxes to indicate where patients should tick each item.

The questionnaire may be adapted for use on-line or by telephone.

The Patient-Specific Functional Scale

This useful questionnaire can be used to quantify activity limitation and measure functional outcome for patients with any orthopaedic condition.

Clinician to read and fill in below: Complete at the end of the history and prior to physical examination.

Initial Assessment:

I am going to ask you to identify up to three important activities that you are unable to do or are having difficulty with as a result of your __________________ problem. Today, are there any activities that you are unable to do or having difficulty with because of your __________________ problem? (Clinician: show scale to patient and have the patient rate each activity).

Follow-up Assessments:

When I assessed you on (state previous assessment date), you told me that you had difficulty with (read all activities from list at a time). Today, do you still have difficulty with: (read and have patient score each item in the list)?

Patient-specific activity scoring scheme (Point to one number):

0 1 2 3 4 5 6 7 8 9 10

Unable to perform activity — Able to perform activity at the same level as before injury or problem

(Date and Score)

Activity	Initial					
1.						
2.						
3.						
4.						
5.						
Additional						
Additional						

Total score = sum of the activity scores/number of activities
Minimum detectable change (90%CI) for average score = 2 points
Minimum detectable change (90%CI) for single activity score = 3 points

PSFS developed by: Stratford, P., Gill, C., Westaway, M., & Binkley, J. (1995). Assessing disability and change on individual patients: a report of a patient specific measure. Physiotherapy Canada, 47, 258-263.

Reproduced with the permission of the authors.

PAIN S-E QUESTIONNAIRE (PSEQ)
Nicholas (1989)

NAME: ____ DATE: ______________________

Please rate how **confident** you are that you can do the following things at present, **despite the pain**. To indicate your answer circle one of the numbers on the scale under each item, where 0 = not at all confident and 6 = completely confident.

For example:

0	1	2	(3)	4	5	6
Not at all confident						Completely confident

Remember, this questionnaire is not asking whether or not your have been doing these things, but rather **how confident you are that you can do them at present, despite the pain.**

	Not at all confident						Completely confident
1. I can enjoy things, despite the pain	0	1	2	3	4	5	6
2. I can do most of the household chores (e.g. tidying -up, washing dishes, etc.), despite the pain	0	1	2	3	4	5	6
3. I can socialise with my friends or family members as often as I used to do, despite the pain.	0	1	2	3	4	5	6
4. I can cope with my pain in most situations	0	1	2	3	4	5	6
5. I can do some form of work, despite the pain. ("work" includes housework, paid and unpaid)	0	1	2	3	4	5	6
6. I can still do many of the things I enjoy doing, such as hobbies or leisure activities, despite the pain	0	1	2	3	4	5	6
7. I can cope with my pain without medication.	0	1	2	3	4	5	6
8. I can still accomplish most of my goals in life, despite the pain	0	1	2	3	4	5	6
9. I can live a normal lifestyle, despite the pain	0	1	2	3	4	5	6
10. I can gradually become more active, despite the pain	0	1	2	3	4	5	6

Save Print
